# Challenging the Minimum Effective Antipsychotic Dose During Maintenance: Implications From 10-Year Follow-Up of First Episode Psychosis

**DOI:** 10.3389/fpsyt.2021.714878

**Published:** 2021-09-07

**Authors:** Chen-Chung Liu, Chih-Min Liu, Yi-Ling Chien, Yi-Ting Lin, Ming H. Hsieh, Tzung-Jeng Hwang, Hai-Gwo Hwu

**Affiliations:** ^1^Department of Psychiatry, National Taiwan University Hospital, Taipei, Taiwan; ^2^Department of Psychiatry, College of Medicine, National Taiwan University, Taipei, Taiwan; ^3^Department of Psychiatry, National Taiwan University Hospital Hsin-Chu Branch, Hsin-Chu, Taiwan

**Keywords:** antipsychotics, first episode psychosis, functioning, maintenance, minimum effective dose

## Abstract

**Background:** Contradictory messages regarding the necessity of long-term antipsychotic treatment after first episode psychosis arouse deliberations in clinical practice. We explored if there is an alternative beyond the dichotomy of maintenance treatment and discontinuation of medications.

**Methods:** We conducted a retrospective observational study by reviewing medical records at the study hospital of a cohort of patients since their participation in an early psychosis study starting from 2006, with special interests in patients able to maintain good functioning under treatment with a low antipsychotic dose.

**Results:** Of the 81 patients with first-episode psychosis, 55 patients (67.9%) had follow-up information for longer than 5 years. The majority (*n* = 46, 83.6%) had non-affective psychosis, 20 patients (36.4%) had full-time employment/education by the time of their latest visit; among them, 15 patients received dosage of antipsychotics no more than the minimum effective dose [chlorpromazine equivalent (CPZE) dose, 200 mg/day]. Besides, 10 of 55 patients (18.2%) only received very low dose antipsychotics (CPZE < 50 mg/day) during maintenance, which was significantly correlated to good functioning. Being male, having a history of hospitalization, and being on clozapine therapy were correlated to poorer functioning. Antipsychotic-free status was achieved only in two non-psychotic patients.

**Conclusions:** A substantial proportion of patients could achieve good functioning under low-dose antipsychotic maintenance after first-episode psychosis, even if they could not completely withdraw antipsychotics in the long term. Optimizing the balance between preventing relapse and preserving functioning by fine-tuning antipsychotic dosage during maintenance is a challenge warranting more clinical attention.

## Introduction

The outcomes for patients with schizophrenia have a chance to move toward favorable trajectories if early intervention is timely delivered at their first episode (1–3). In the era of first-generation antipsychotics, treatment response to first episode patients was already known to be much better than that after multiple episodes (4). Even employing a rigorous and uniform definition of “remission” (5), the remission rates could reach 24–78% (6), overall above 50% estimated by a meta-analysis (7), which are much higher than previously believed. While the wide range of remission rates suggests variety by cohort not only in the duration of follow-up (from 1 to 10 years) and the time criteria (persistently remitted for 6 months), patients' medication status and other psychosocial factors might also affect the long-term outcomes. However, review studies revealed that different series of cohorts failed to yield consistent predictors of outcomes after first-episode psychosis (FEP) (8–11).

Medication non-adherence is believed to be the most important predictor of relapse (12). This statement has been supported by several clinical trials examining relapse rates during medication discontinuation (13–17). However, patients still tend to stop medication once their symptoms are remitted (18–20). Benefitting from early antipsychotic intervention, those good and quick responders might think it easy to control their psychotic symptoms as long as they resume antipsychotics in time, once early signs of relapse re-emerge (21). However, such an intermittent treatment strategy is not recommended, as higher dose and longer duration are needed to achieve remission after relapse episodes (22–24), and more of those who have relapses become non-responders (25) and are likely to have more residual symptoms (24). Almost inevitably, early discontinuation of antipsychotics is related to worse long-term outcomes (26, 27). Thus, even though currently there are no uniform guidelines for the duration of continuous treatment after a single psychotic episode (28, 29), a critical review appraising the benefit-to-risk ratio endorsed long-term antipsychotic treatment (30).

The aforementioned opinion has gained strong support from some renowned researchers who asserted the outweighed benefits of continuous antipsychotic treatment (31, 32), but doubts were cast by those who valued adjunctive psychosocial intervention as just important as pharmacological intervention, voicing their concerns about potential dose-dependent adverse effects, impaired cognitive functioning, and even the toxic effect on the brain of long-term use of antipsychotics (33–37). Thus, the benefit of prevention from relapse by antipsychotics must be cautiously calibrated (38). Even though the quality of longitudinal follow-up studies has been questioned by those who advocate for continuous antipsychotic treatment, the fact that a substantial proportion of patients could lead their lives with adequate functioning, even though not all were in a remitted state or were maintained on antipsychotic treatment, instills a hope in patients recently recovering from their first-episode psychosis (39–43).

Indeed, in the real world, not only do patients wish to discontinue treatment after FEP (21), but a substantial proportion of clinicians are also thinking of discontinuing antipsychotic medication following symptom remission after FEP (44, 45). In a qualitative study with an in-depth look into a few cases in this group, we found that diagnostic stability, treatment preferences, protective or perpetuating psychosocial factors, individual coping strategies, and personalized formulation of illness all contributed to patients' trajectories after FEP; while antipsychotic medication was really helpful and essential when patients were facing challenges and crises, it was not perceived to play a pivotal role once they were in a relatively non-eventful period of life (46).

To explore if there can be a compromise between maintenance and discontinuation of antipsychotics in the long-term treatment after a first psychotic episode, we retrieved the medical records of a cohort of patients who participated in our early psychosis project from 2006 to 2010. We tried to (1) identify if certain patients could be maintained by an antipsychotic dose below the currently recognized minimum effective dose (MED) (47), (2) examine if being treated with a low antipsychotic dose is correlated to better functioning, and (3) describe those who could do well under a very low dose qualitatively.

## Methods

### Participants

This retrospective naturalistic observational study focused on a cohort of 81 subjects, comprised of 60 patients with FEP at recruitment (the FEP group) of a follow-up study of the psychopathological progress of early schizophrenia-like disorder (the SOPRES study in Taiwan) and 21 of the 59 patients who initially presented as in an ultrahigh-risk state (UHR) converting into FEP during the first 2 years of the SOPRES follow-up (the UHR+ group) (48). This study was approved by the Research Ethics Committee of the study hospital (REC201811051RINA).

### Measures

#### Data Extraction

Patients' medical records of outpatient, inpatient, and emergency department visits at the study hospital from 2006 to 2019 were retrieved electronically and manually. A checklist was developed for data coding, including duration of follow-up, history of hospitalization, occupational functioning, medication status (whether receiving clozapine or under long-acting injectable antipsychotic treatment), estimated daily antipsychotic dose by converting to chlorpromazine equivalent (CPZE) (47, 49), and revision of diagnosis, if any. As rating scales were not routinely employed in clinical practice, these data were derived by extracting messages read from doctor's records and summarized in individual field notes. The majority of patients were followed by board-certified psychiatrists of the SOPRES research team, so we could reach consensus upon any uncertainty regarding data extraction by direct discussion with the patient's attending psychiatrist. If the patient was not attended by a member of our research team, we tried to verify the information by consulting with his or her primary attending psychiatrist, if available.

#### Supplement Information Regarding the Early Lost-To-Follow-Up Patients

For those who had no information available from medical records since SOPRES follow-up ended in 2010, a carefully worded letter was sent to invite them to participate in follow-up interviews. Details of this approach, including how to comply with ethical codes on such a sensitive issue, were addressed elsewhere (46). This approach allowed us to reach patients whose records were not updated in our medical records during 2011–2015, to conduct in-depth qualitative interviews, and to get descriptions of how they have been doing with their illness after the SOPRES study ended. In fact, a few patients returned to be followed up at the study hospital after the invitation. These data were also incorporated into this analysis.

#### Defining Functioning

A patient's functioning status was defined as follows: those who had full employment or were in a full educational program (with >15 h/week for more than 3 months during the past 6 months) during their latest follow-up year were designated as having good functioning; those who participated in work or education less than that intensity, in a rehabilitation program/sheltering work, or were able to regularly help doing chores at home were designated as having partial functioning; the remaining were in the limited functioning group.

### Statistical Analysis

Descriptive statistics were used to display the proportions of patients fitting each category of interest. Clinical characteristics were examined for any significant differences between groups (UHR+ vs. FEP) and among functioning levels (good vs. partial vs. limited) by chi-square statistics for categorical variables and by *t*-test or ANOVA for continuous variables. The probability of type I error was set at 0.05 and the power at 0.8; the estimated sample size for comparison of a 0.4 (0.5 vs. 0.1) difference in proportions between groups was 17 in each group (http://powerandsamplesize.com/Calculators). Logistic regression analysis with backward selection to remove variables with a probability >0.2 was employed to test whether any significant predictors of good or limited functioning remained after taking into account other variables. A two-sided *p* < 0.05 was considered to be statistically significant.

### Qualitative Presentations

A few cases bearing unique characteristics and trajectories worthy of in-depth discussion were described briefly in this report, with patients' confidentiality and anonymity being rigorously protected and no identifiable personal data revealed.

## Results

### Overall Participants for Follow-Up

The characteristics of patients are displayed in Table 1. They were generally young at recruitment, as our inclusion criteria of age was between 16 and 32 years by then (50). Twelve patients did not return to follow-up after 1 year (average 0.4 ± 0.3 years), 14 patients had follow-up durations between 1 and 5 years (2.6 ± 1.1 years), and 55 patients had records at the study hospital for more than 5 years (9.9 ± 2.0 years). There was no significant difference in baseline age and gender distribution between those who had follow-up longer than 5 years and those who did not.

**Table 1:** Summary of demographic and clinical features of the original cohort.

	**UHR+ (*n* = 21)**	**FEP (*n* = 60)**	**Total (*n* = 81)**
Age at recruitment, years	20.6 ± 3.6	21.1 ± 4.2	21.0 ± 4.0
Gender male (%)	10 (47.6)	26 (43.3)	36 (44.4)
Duration of follow-up			
<1 year 1–5 years >5 years	0 4 17	12 10 38	12 14 55
Diagnosis			
Schizophrenia Schizophreniform Schizoaffective disorder, depressive Schizoaffective disorder, bipolar Bipolar disorder Psychotic depression Brief psychotic disorder Schizotypal disorder Non-psychotic disorder	15 (71.4) 2 (9.5) 0 (0) 0 (0) 1 (4.8) 0 (0) 1 (4.8) 0 (0) 2 (9.5)	42 (70) 2 (3.3) 1 (1.7) 4 (6.7) 2 (3.3) 3 (5) 2 (3.3) 3 (5) 1 (1.7)	57 (70.4) 4 (4.9) 1 (1.2) 4 (4.9) 3 (3.7) 3 (3.7) 3 (3.7) 3 (3.7) 3 (3.7)

*UHR+, ultra-high risk subjects converting to first episode psychosis after recruitment; FEP, first episode psychosis. The counts in each cell are the number of subjects and (%)*.

### Participants With Follow-Up Records for More Than 5 Years

Focused on those who had follow-up information for more than 5 years (Table 2), there were no significant differences in age, gender, length of follow-up, distribution of diagnosis, functioning level, or treatment modalities between those who converted to FEP after recruitment (UHR+ group) and those who were already in FEP at recruitment (FEP group). By the time of censoring, 20 (36.4%) were categorized as good functioning, 16 (29.1%) with partial functioning, and 19 (34.5%) with limited functioning. Their baseline and follow-up characteristics were comparable to each other.

**Table 2:** Clinical characteristics of patients with follow-up for more than 5 years.

	**Total (*n* = 55)**	**UHR +(*n* = 17)**	**FEP (*n* = 38)**	***p***
Age at recruitment, years old (Mean ± SD)	20.9 ± 4.2	20.5 ± 3.8	21.0 ± 4.5	0.691
Gender, Male	22 (40)	7 (41.2)	15 (39.5)	0.905
Length of follow-up years (Mean ± SD)	9.88 ± 2.00	10.08 ± 1.97	9.79 ± 2.03	0.615
Diagnosis^†^				
Non-affective psychosis Affective psychosis Brief psychotic disorder Schizotypal disorder Non-psychotic disorder	46 (83.6) 5 (9.1) 1 (1.8) 1 (1.8) 2 (3.6)	15 (88.2) 1 (5.9) 0 0 1 (5.9)	31 (81.6) 4 (10.5) 1 (2.6) 1 (2.6) 1 (2.6)	0.813
Functioning				
Good Partial Limited	20 (36.4) 16 (29.1) 19 (34.5)	8 (47.1) 3 (17.6) 6 (35.3)	12 (31.6) 13 (34.2) 13 (34.2)	0.390
Never hospitalized	28 (50.9)	7 (42.1)	21 (55.3)	0.334
On LAI antipsychotic treatment	6 (10.9)	3 (17.6)	3 (7.9)	0.284
On clozapine therapy	8 (14.5)	3 (17.6)	5 (13.2)	0.663
Antipsychotics CPZE < 200 mg/d	17 (30.9)	7 (41.2)	10 (26.3)	0.270
Antipsychotics CPZE < 50 mg/d	10 (18.2)	4 (23.5)	6 (15.8)	0.492

† *Non-affective psychosis includes schizophrenia, schizophreniform, and schizoaffective disorder, depressive type; Affective psychosis includes shizoaffective disorder, bipolar type, bipolar disorder, psychotic depression. UHR+, ultra-high risk subjects converting to first episode psychosis after recruitment; FEP, first episode psychosis; SD, standard deviation; CPZE, chlorpromazine equivalent; LAI, long-acting injectable. The counts in each cell are the number of subjects and (%), except for the two denoted as Mean ± SD*.

### Antipsychotic Dose and Other Factors Related to Long-Term Functioning

Comparing patients' characteristics between functioning levels as shown in Table 3, significantly more male patients were ranked with limited functioning. The majority (75%) of good functioning patients received antipsychotic dose no more than the MED (CPZE 200 mg/day), including eight patients under a dose as low as CPZE 50 mg/day, in contrast to only two patients with partial functioning and none of the patients with limited functioning who were treated at such a low dose level. Statistically, both low dose (CPZE < 200 mg/day) and very low dose (CPZE < 50 mg/day) groups were significantly related to good functioning.

**Table 3:** Factors related to functioning during long-term follow-up.

	**Good (*n* = 20)**	**Partial(*n* = 16)**	**Limited (*n* = 19)**	***p***
Age at recruitment, years	22.4 ± 5.0	19.9 ± 4.0	20.1 ± 3.1	0.146
Length of follow-up, years	10.6 ± 1.5	9.9 ± 2.0	9.1 ± 2.3	0.072
Gender (Male)	6 (30.0)	4 (25.0)	12 (63.2)	0.037
UHR+	8 (40.0)	3 (18.8)	6 (31.6)	0.390
Diagnosis				
Non-Affective psychosis Affective psychosis Other^†^	16 (80.0) 2 (10.0) 2 (10.0)	12 (75.0)2 (12.5)2 (12.5)	18 (94.7) 1 (5.3) 0 (0)	0.526
Never hospitalized	13 (65.0)	9 (56.3)	6 (31.6)	0.100
On clozapine	1 (5)	1 (6.3)	6 (31.6)	0.034
On LAIA	2 (10.0)	2 (12.5)	2 (10.5)	0.970
CPZE < 200 mg/d	15 (75.0)	2 (12.5)	0 (0)	<0.001
CPZE < 50 mg/d	8 (40.0)	2 (12.5)	0 (0)	0.003

† *Other diagnoses include brief psychotic disorder, schizotypal disorder, and non-psychotic disorder. UHR+, ultra-high risk subjects converting to first episode psychosis after recruitment; CPZE, chlorpromazine equivalent; LAIA, long-acting injectable antipsychotic*.

In addition to antipsychotic dose, there was a trend of better functioning in patients who had never been hospitalized, and logistic regression analysis revealed that history of admission during the disease course was a predictor of limited functioning [odds ratio (OR), 12.35; 95% confidence interval (CI), 1.35–113.1; *p* = 0.026]. Being on clozapine therapy seemed to be related to limited functioning (*p* = 0.034), but the impact became less significant when the variables of age and male gender were taken into account (OR, 6.39; 95% CI, 0.91–44.78; *p* = 0.062). Treatment with a long-acting injectable antipsychotic was not related to functioning level. We also found older age at recruitment correlated with a higher chance of having good functioning (OR, 1.21; 95% CI, 1.03–1.42, *p* = 0.017). Even after excluding those two antipsychotic-free non-psychotic patients from the very low dose group and taking into account the aforementioned clinical variables examined by logistic regression analysis, the relationship between treatment with CPZE < 50 mg/day and good functioning persisted (OR, 20.6; 95% CI, 2.45–174.1, *p* = 0.004).

As the majority of the patients (*n* = 46, 83.6%) had schizophrenia spectrum disorder but only a scattered number of patients in other categories, we did not compare functioning level based on diagnoses quantitatively.

### Focused on Patients Treated With a Very Low Antipsychotic Dose

Table 4 reports those 10 patients with good functioning under a very low dose antipsychotic treatment. Five of them were diagnosed with schizophrenia-spectrum disorder, all having experiences of relapse or recurrence of symptoms warning of a relapse when they stopped all antipsychotics. All responded well soon after resuming antipsychotic treatment. Giving highlights to two extreme cases, one patient who had discontinued medication completely for 10 years, and then returned to visit with recurrence of full-blown psychosis, again responded well to very low dose antipsychotics like she did in her first episode. The other patient had recurrent brief psychotic episodes almost annually, with good response to 1–2 week higher dose of aripiprazole (2.5–5 mg/day) and then tapered down to below 2.5 mg/day once her psychotic symptoms remitted because of her concern about medication-related oversedation. The patient tried to discontinue such a very low dose intermittent maintenance but never achieved this goal. Her diagnosis could be schizophrenia, multiple episodes with full remission, or recurrent brief psychotic disorder, but most importantly her functioning could be maintained quite well during the remitted states most of her life.

**Table 4:** Summary of patients receiving only very-low-dose or no antipsychotic treatment during maintenance.

**Age^†^**	**Sex**	**F/U (years)**	**Diagnosis**	**Medication status**	**Functioning**	**Remarks**
38	F	11.7	Schizophrenia	Aripiprazole 2.5 mg/d, tapering down by herself	Full-time job all along the course, even during psychosis	Good responder
39	F	11.7	Schizophrenia	Aripiprazole 2.5 mg/d or lower, intermittent use	Full time job most of time	Good responder
29	M	10.6	Schizophrenia	Trifluoperazine 2.5 mg/d	Full-time job in recent 4 years	Sensitive to effect and AEs of aripiprazole
30	F	10.1	Schizophrenia	Quetiapine 50 mg/d plus escitalopram 5 mg/d	Full-time student after simplifying treatment to current regimen in in recent 2 years	Might have treated her neuropsychiatric AEs related to other antipsychotics as psychotic symptoms during the course
29	M	9.2	Schizophreniform disorder	Aripiprazole 2.5 mg/d	Full-time education/job along the course	Good responder
31	F	8.6	Dysthymia; history of dissociation	Antipsychotic-free > 7 years	Full- or part-time jobs most of time	Revision of diagnosis; recovered under good supportive system
26	M	7	Schizotypal disorder and OCD	Mainly maintained by ADs for OCD and depression	Long-term underachieved status	Partial response to various combinations of pharmacotherapy
23	M	5.7	Bipolar disorder	Intermittent use of quetiapine 25 mg/d	Full-time student with adequate performance	A transient psychotic episode only, not the main concern during the course
21	M	5.2	Dysthymia	Only treated with antipsychotic for 1 month	Long-term underachieved status	Revision of diagnosis; alleging long-term lack of support from family
28	F	12	Brief psychotic disorder	Aripiprazole 1.25 to 2.5 mg/d, up to 5 mg/d in an episode for 1–2 weeks	Full-time job most of time, only needed to take a 1–2 week sick leave during each episode	Each episode precipitated by an identifiable stressor; either recurrent brief psychosis or good responder of schizophrenia

† *Age in this Table denotes the patient's age at latest visit. AD, antidepressant; AEs, adverse effects; F/U, follow-up; OCD, obsessive compulsive disorder*.

### Focused on Patients With Good Functioning

In addition to those 8 patients with good functioning under very low dose treatment, the other 12 good-functioning patients included 11 with schizophrenia and 1 with schizoaffective disorder bipolar type. Seven of the 12 were maintained on an antipsychotic dose range of CPZE 50–200 mg/day, including two patients not meeting remission criteria, but who opted to use an irregular dosing schedule to achieve a balance between effects and side effects, as did some patients reported by Gaebel et al. (13). Tallied together, a total of 15 out of 20 (75%) good-functioning patients were maintained under no more than CPZE 200 mg/day, comprising 27.3% (15/55) patients of this cohort.

## Discussion

### The Minimum Effective Dose for Stable Patients: An Unexplored Question

It is time to reconsider the use of antipsychotics beyond the dichotomy of maintenance and discontinuation for remitted psychosis (51), and the applicability of the minimum effective dose based on comparing antipsychotics to placebo in randomized, double-blind, fixed-dose trials to stable patients (47, 49). Our results were consistent with a recent review article that suggests that reducing antipsychotics to above CPZE 200 mg/day (the designated minimum effective dose) did not increase the risk of relapse compared to being maintained at a higher dose (52). Moreover, exploring the even lower dose range provides valuable information to modify the impression regarding the role of antipsychotic treatment during the long-term course of psychosis. Even though dose-reduction trials have shown an increase risk of relapse if medication is reduced to a very low dose level (53), such a result might be caused either by reducing too much (for example, half dose) at a time (30) or by conducting stepwise dose reduction after a relatively short period of stabilization (for example, every 1–3 months) at each dose level (54). Tapering antipsychotic treatment very slowly and in a hyperbolic manner was suggested by Horowitz et al. (55), as to allow neuroadapations of dopamine D2 receptors and minimize the risk of dopamine-supersensitivity psychosis (56).

Our results revealed that, in real world practice, low-dose antipsychotic treatment was not uncommon in patients with good functioning. Tapering down medication may alleviate dose-dependent adverse effects of antipsychotics, especially neurocognitive impairment and somnolence (37), a possible explanation of their improved outcomes. Patients could reduce dosage with a slower tempo and wait for better timing (such as when they were not under significant psychosocial stressors) after a longer stabilization period before the next dose reduction to avoid the negative impact of dopamine supersensitivity. Consequently, reducing to a very low dose level is attainable, and its benefits might outweigh its risks in the long term.

### Potential Mislabeling Patients Treated With a Very Low but Effective Dose as Patients Received No Medication

In the report by Wunderink et al. of their controversial findings of outcomes between 18 months and 7 years (41), patients receiving a daily equivalent dose of haloperidol <1 mg/day (or CPZE <50 mg/day) were categorized as the discontinuation group, and this group had better functioning compared to their maintenance counterpart. We argue that some patients in their discontinuation group might be like the cases who received a very low dose maintenance and exhibited good functioning in our cohort, comprising 14.5% (8/55) or at least 9.9% (8/81). Indeed, a series of studies have challenged that the therapeutic window of dopamine D2 receptor blockade in the maintenance phase could be lower than 65% (57–61), and two clinical trials showed improved cognitive functioning after reducing the dose of antipsychotics in stable patients (62, 63), which might contribute to the good functioning of our cohort.

On the other hand, in our cohort, the majority of good-functioning, very-low-dose patients have experienced impending or even a full-blown relapse if they withdrew antipsychotics completely, except for the two patients who were eventually rediagnosed as non-psychotic. Thus, a very-low-dose antipsychotic treatment still provided the protection to prevent from psychotic relapse. Hence, a fine-tuning of dosage to achieve the lowest effective antipsychotic dose during maintenance, even lower than the previously recognized minimum, will be the key to optimize the balance between preventing relapse and preserving functioning.

### Rediagnosis After First Episode Psychosis Leading to Revision of Medication Use

The distribution and stability of diagnosis of our study cohort are comparable to those of a previous report (64) and the results of the meta-analysis by Fusar-Poli et al. (65). Inevitably, a few patients recruited in an era when heightened attention was aroused to focus on early psychosis and pre-psychotic states might have been mislabeled as FEP during the early phase of our study. One of the three non-psychotic patients did well during follow-up, which she attributed to her sound supportive system (46), while the other two led a demoralized life with suboptimal performance, suggesting that the role of pharmacotherapy was not the key to their functioning. Patients in this category also need help but unlikely would they be benefited from long-term antipsychotic treatment. Furthermore, all three patients had their onset of psychotic symptoms as teenagers but exhibited predominantly depressive symptoms rather than psychosis in the long run, in line with a longitudinal study that followed psychotic-like and depressive symptomatology in twin adolescents (66). Thus, it is worthwhile to remember that rediagnosis at around 1 year after a diagnosis of FEP should have been included in the treatment guidelines (22).

## Limitations

There are a few limitations to address in a naturalistic observational study. First, one-third of this cohort was missing after 5 years, an attritional rate comparable to previous long-term follow-up studies. Second, only the medical records of the study hospital were retrieved, even though some patients have been invited to take in-depth interviews. Third, remission status was not formally assessed, and patients' functioning level was mainly reached by consensus of the study team. Fourth, limited information was available regarding medication adherence, and the actual dose taken by the individual patient was not verified except for a few with qualitative information. Fifth, the sample size is not big enough to allow sophisticated statistical analysis; also, the relationship between the use of clozapine, hospitalization, and poor functioning was more likely a result of confounding by severity or confounding by indication. Sixth, no information regarding supportive system and only sketchy descriptions of psychosocial stressors could be extracted from the records; both are important factors in maintaining a remission and good functioning. Nonetheless, we have tried to utilize every piece of information and avoid drawing inferences from uncertain data.

## Perspectives into the Future

Echoing the notion that “Less (antipsychotic) Is More (benefiting)” in terms of pharmacotherapy following remission from the first episode of psychosis proposed by McGorry et al. (67), our evidence suggests that at least a substantial proportion of patients could do well under a low, even a very low, dose of antipsychotic medication. Inspired by such encouraging clinical observations, we proposed a protocol to conduct a carefully guided antipsychotic dose-reduction trial for patients remitted from psychosis (68). We anticipate gaining more solid evidence to consider revising the schizophrenia long-term treatment guidelines to include “on when and how slowly to reduce antipsychotics, and in whom it is appropriate to eventually stop them” (69). Our hope is that, eventually, we can invite patients to participate in shared decision-making regarding the optimal use of antipsychotics (70) to help them achieve better outcomes in every aspect of their lives.

## Data Availability Statement

The raw data supporting the conclusions of this article will be made available by the authors, without undue reservation.

## Ethics Statement

The studies involving human participants were reviewed and approved by Research Ethics Committee of the National Taiwan University Hospital. The patients/participants have provided their written informed consent to participate in the previous SOPRES study.

## Author Contributions

C-CL, C-ML, and H-GH reviewed the literature and designed the study. C-CL wrote the first draft. All authors contributed to and have approved the final manuscript, participated in the verification, interpretation, and discussion of data.

## Funding

This work was supported by research grants NHRI-EX95, 96, 97, 98, 99-9511PP, PI H-GH from the National Health Research Institutes, Taiwan for the SOPRES study; NTUH 103-S2432, NTUH 104-S2772, PI C-CL from National Taiwan University Hospital research grants for review of medical records; and MOST 106-2314-B-002-106 and MOST 107-2314-B-002-222-MY3, PI C-CL from the Ministry of Science and Technology, Taiwan for the preparation of this paper. The funding sources did not participate in the preparation, review, approval, or decision to submit this manuscript for publication.

## Conflict of Interest

The authors declare that the research was conducted in the absence of any commercial or financial relationships that could be construed as a potential conflict of interest.

## Publisher's Note

All claims expressed in this article are solely those of the authors and do not necessarily represent those of their affiliated organizations, or those of the publisher, the editors and the reviewers. Any product that may be evaluated in this article, or claim that may be made by its manufacturer, is not guaranteed or endorsed by the publisher.

## References

[1] Keshavan MS Haas G Miewald J Montrose DM Reddy R Schooler NR. Prolonged untreated illness duration from prodromal onset predicts outcome in first episode psychoses. Schizophr Bull. (2003) 29:757–69. 10.1093/oxfordjournals.schbul.a007045 14989413

[2] Larsen TK McGlashan TH Johannessen JO Friis S Guldberg C Haahr U. Shortened duration of untreated first episode of psychosis: changes in patient characteristics at treatment. Am J Psychiatry. (2001) 158:1917–9. 10.1176/appi.ajp.158.11.1917 11691702

[3] Malla AK Norman RM Manchanda R Ahmed MR Scholten D Harricharan R. One year outcome in first episode psychosis: influence of DUP and other predictors. Schizophr Res. (2002) 54:231–42. 10.1016/S0920-9964(01)00254-7 11950548

[4] Lieberman JA Alvir JM Koreen A Geisler S Chakos M Sheitman B. Psychobiologic correlates of treatment response in schizophrenia. Neuropsychopharmacology. (1996) 14:13S–21. 10.1016/0893-133X(95)00200-W 8866739

[5] Andreasen NC Carpenter WT Jr Kane JM Lasser RA Marder SR Weinberger DR. Remission in schizophrenia: proposed criteria and rationale for consensus. Am J Psychiatry. (2005) 162:441–9. 10.1176/appi.ajp.162.3.441 15741458

[6] Chang WC Chan TC Chen ES Hui CL Wong GH Chan SK. The concurrent and predictive validity of symptomatic remission criteria in first-episode schizophrenia. Schizophr Res. (2013) 143:107–15. 10.1016/j.schres.2012.10.016 23151398

[7] Lally J Ajnakina O Stubbs B Cullinane M Murphy KC Gaughran F. Remission and recovery from first-episode psychosis in adults: systematic review and meta-analysis of long-term outcome studies. Br J Psychiatry. (2017) 211:350–8. 10.1192/bjp.bp.117.201475 28982659

[8] Bowtell M Eaton S Thien K Bardell-Williams M Downey L Ratheesh A. Rates and predictors of relapse following discontinuation of antipsychotic medication after a first episode of psychosis. Schizophr Res. (2018) 195:231–6. 10.1016/j.schres.2017.10.030 29066258

[9] Alvarez-Jimenez M Gleeson JF Henry LP Harrigan SM Harris MG Amminger GP. Prediction of a single psychotic episode: a 7.5-year, prospective study in first-episode psychosis. Schizophr Res. (2011) 125:236–46. 10.1016/j.schres.2010.10.020 21081266

[10] Bowtell M Ratheesh A McGorry P Killackey E O'Donoghue B. Clinical and demographic predictors of continuing remission or relapse following discontinuation of antipsychotic medication after a first episode of psychosis. A systematic review. Schizophr Res. (2018) 197:9–18. 10.1016/j.schres.2017.11.010 29146020

[11] Hui CL Honer WG Lee EH Chang WC Chan SK Chen ES. Predicting first-episode psychosis patients who will never relapse over 10 years. Psychol Med. (2019) 49:2206–14. 10.1017/S0033291718003070 30375301

[12] Robinson D Woerner MG Alvir JM Bilder R Goldman R Geisler S. Predictors of relapse following response from a first episode of schizophrenia or schizoaffective disorder. Arch Gen Psychiatry. (1999) 56:241–7. 10.1001/archpsyc.56.3.241 10078501

[13] Gaebel W Riesbeck M Wolwer W Klimke A Eickhoff M von Wilmsdorff M. Relapse prevention in first-episode schizophrenia—maintenance vs. intermittent drug treatment with prodrome-based early intervention: results of a randomized controlled trial within the German research network on Schizophrenia. J Clin Psychiatry. (2011) 72:205–18. 10.4088/JCP.09m05459yel 20673559

[14] Wunderink L Nienhuis FJ Sytema S Slooff CJ Knegtering R Wiersma D. Guided discontinuation versus maintenance treatment in remitted first-episode psychosis: relapse rates and functional outcome. J Clin Psychiatry. (2007) 68:654–61. 10.4088/JCP.v68n0502 17503973

[15] Chen EY Hui CL Lam MM Chiu CP Law CW Chung DW. Maintenance treatment with quetiapine versus discontinuation after one year of treatment in patients with remitted first episode psychosis: randomised controlled trial. BMJ. (2010) 341:c4024. 10.1136/bmj.c4024 20724402 PMC2924475

[16] Emsley R Oosthuizen PP Koen L Niehaus DJ Martinez G. Symptom recurrence following intermittent treatment in first-episode schizophrenia successfully treated for 2 years: a 3-year open-label clinical study. J Clin Psychiatry. (2012) 73:e541–7. 10.4088/JCP.11m07138 22579160

[17] Caseiro O Perez-Iglesias R Mata I Martinez-Garcia O Pelayo-Teran JM Tabares-Seisdedos R. Predicting relapse after a first episode of non-affective psychosis: a three-year follow-up study. J Psychiatr Res. (2012) 46:1099–105. 10.1016/j.jpsychires.2012.05.001 22721546

[18] Kahn RS Fleischhacker WW Boter H Davidson M Vergouwe Y Keet IP. Effectiveness of antipsychotic drugs in first-episode schizophrenia and schizophreniform disorder: an open randomised clinical trial. Lancet. (2008) 371:1085–97. 10.1016/S0140-6736(08)60486-9 18374841

[19] McEvoy JP Lieberman JA Perkins DO Hamer RM Gu H Lazarus A. Efficacy and tolerability of olanzapine, quetiapine, and risperidone in the treatment of early psychosis: a randomized, double-blind 52-week comparison. Am J Psychiatry. (2007) 164:1050–60. 10.1176/ajp.2007.164.7.1050 17606657

[20] Cooper D Moisan J Gregoire JP. Adherence to atypical antipsychotic treatment among newly treated patients: a population-based study in schizophrenia. J Clin Psychiatry. (2007) 68:818–25. 10.4088/JCP.v68n0601 17592904

[21] Perkins DO Gu H Weiden PJ McEvoy JP Hamer RM Lieberman JA. Predictors of treatment discontinuation and medication nonadherence in patients recovering from a first episode of schizophrenia, schizophreniform disorder, or schizoaffective disorder: a randomized, double-blind, flexible-dose, multicenter study. J Clin Psychiatry. (2008) 69:106–13. 10.4088/JCP.v69n0114 18312044

[22] Barnes TR. Evidence-based guidelines for the pharmacological treatment of schizophrenia: recommendations from the British Association for Psychopharmacology. J Psychopharmacol. (2011) 25:567–620. 10.1177/0269881110391123 21292923

[23] De Hert M Sermon J Geerts P Vansteelandt K Peuskens J Detraux J. The use of continuous treatment versus placebo or intermittent treatment strategies in stabilized patients with schizophrenia: a systematic review and meta-analysis of randomized controlled trials with first- and second-generation antipsychotics. CNS Drugs. (2015) 29:637–58. 10.1007/s40263-015-0269-4 26293744

[24] Takeuchi H Siu C Remington G Fervaha G Zipursky RB Foussias G. Does relapse contribute to treatment resistance? Antipsychotic response in first- vs. second-episode schizophrenia. Neuropsychopharmacology. (2019) 44:1036–42. 10.1038/s41386-018-0278-3 30514883 PMC6462044

[25] Emsley R Nuamah I Hough D Gopal S. Treatment response after relapse in a placebo-controlled maintenance trial in schizophrenia. Schizophr Res. (2012) 138:29–34. 10.1016/j.schres.2012.02.030 22446143

[26] Hui CLM Honer WG Lee EHM Chang WC Chan SKW Chen ESM. Long-term effects of discontinuation from antipsychotic maintenance following first-episode schizophrenia and related disorders: a 10 year follow-up of a randomised, double-blind trial. Lancet Psychiatry. (2018) 5:432–42. 10.1016/S2215-0366(18)30090-7 29551618

[27] Tiihonen J Tanskanen A Taipale H. 20-year nationwide follow-up study on discontinuation of antipsychotic treatment in first-episode schizophrenia. Am J Psychiatry. (2018) 175:765–73. 10.1176/appi.ajp.2018.17091001 29621900

[28] Kane JM Garcia-Ribera C. Clinical guideline recommendations for antipsychotic long-acting injections. Br J Psychiatry. (2009) 195:S63–7. 10.1192/bjp.195.52.s63 19880920

[29] Takeuchi H Suzuki T Uchida H Watanabe K Mimura M. Antipsychotic treatment for schizophrenia in the maintenance phase: a systematic review of the guidelines and algorithms. Schizophr Res. (2012) 134:219–25. 10.1016/j.schres.2011.11.021 22154594

[30] Correll CU Rubio JM Kane JM. What is the risk-benefit ratio of long-term antipsychotic treatment in people with schizophrenia? World Psychiatry. (2018) 17:149–60. 10.1002/wps.20516 29856543 PMC5980517

[31] Fleischhacker WW. The long-term treatment of schizophrenia with antipsychotics: a perennial debate. World Psychiatry. (2018) 17:169–70. 10.1002/wps.20542 29856566 PMC5980630

[32] Lieberman JA. Disease modifying effects of antipsychotic drugs in schizophrenia: a clinical and neurobiological perspective. World Psychiatry. (2018) 17:163–5. 10.1002/wps.20543 29856557 PMC5980457

[33] Harrow M Jobe TH. Long-term antipsychotic treatment of schizophrenia: does it help or hurt over a 20-year period? World Psychiatry. (2018) 17:162–3. 10.1002/wps.20518 29856562 PMC5980604

[34] Isohanni M Miettunen J Jaaskelainen E Moilanen J Hulkko A Huhtaniska S. Under-utilized opportunities to optimize medication management in long-term treatment of schizophrenia. World Psychiatry. (2018) 17:172–3. 10.1002/wps.20523 29856540 PMC5980385

[35] Marder SR Zito MF. Will I need to take these medications for the rest of my life? World Psychiatry. (2018) 17:165–6. 10.1002/wps.20519 29856554 PMC5980574

[36] Ho BC Andreasen NC Ziebell S Pierson R Magnotta V. Long-term antipsychotic treatment and brain volumes: a longitudinal study of first-episode schizophrenia. Arch Gen Psychiatry. (2011) 68:128–37. 10.1001/archgenpsychiatry.2010.199 21300943 PMC3476840

[37] Yoshida K Takeuchi H. Dose-dependent effects of antipsychotics on efficacy and adverse effects in schizophrenia. Behav Brain Res. (2021) 402:113098. 10.1016/j.bbr.2020.113098 33417992

[38] Leucht S Tardy M Komossa K Heres S Kissling W Salanti G. Antipsychotic drugs versus placebo for relapse prevention in schizophrenia: a systematic review and meta-analysis. Lancet. (2012) 379:2063–71. 10.1016/S0140-6736(12)60239-6 22560607

[39] Austin SF Mors O Secher RG Hjorthoj CR Albert N Bertelsen M. Predictors of recovery in first episode psychosis: the OPUS cohort at 10 year follow-up. Schizophr Res. (2013) 150:163–8. 10.1016/j.schres.2013.07.031 23932664

[40] Morgan C Lappin J Heslin M Donoghue K Lomas B Reininghaus U. Reappraising the long-term course and outcome of psychotic disorders: the AESOP-10 study. Psychol Med. (2014) 44:2713–26. 10.1017/S0033291714000282 25066181 PMC4134320

[41] Wunderink L Nieboer RM Wiersma D Sytema S Nienhuis FJ. Recovery in remitted first-episode psychosis at 7 years of follow-up of an early dose reduction/discontinuation or maintenance treatment strategy: long-term follow-up of a 2-year randomized clinical trial. JAMA Psychiatry. (2013) 70:913–20. 10.1001/jamapsychiatry.2013.19 23824214

[42] Harrow M Jobe TH Faull RN Yang J. A 20-Year multi-followup longitudinal study assessing whether antipsychotic medications contribute to work functioning in schizophrenia. Psychiatry Res. (2017) 256:267–74. 10.1016/j.psychres.2017.06.069 28651219 PMC5661946

[43] Wils RS Gotfredsen DR Hjorthoj C Austin SF Albert N Secher RG. Antipsychotic medication and remission of psychotic symptoms 10years after a first-episode psychosis. Schizophr Res. (2017) 182:42–8. 10.1016/j.schres.2016.10.030 28277310

[44] Thompson A Singh S Birchwood M. Views of early psychosis clinicians on discontinuation of antipsychotic medication following symptom remission in first episode psychosis. Early Interv Psychiatry. (2015) 10:355–61. 10.1111/eip.12244 25962446

[45] Hui CL Wong AK Leung WW Lee EH Chan SK Chang WC. Psychiatrists' opinion towards medication discontinuation in remitted first-episode psychosis: a multi-site study of the Asian network for early psychosis. Early Interv Psychiatry. (2018) 13:1329–37. 10.1111/eip.12765 30485671

[46] Liu CC Lin YT Liu CM Hsieh MH Chien YL Hwang TJ. Trajectories after first-episode psychosis: complement to ambiguous outcomes of long-term antipsychotic treatment by exploring a few hidden cases. Early Interv Psychiatry. (2019) 13:895–901. 10.1111/eip.12696 29927087

[47] Woods SW. Chlorpromazine equivalent doses for the newer atypical antipsychotics. J Clin Psychiatry. (2003) 64:663–7. 10.4088/JCP.v64n0607 12823080

[48] Liu CC Lai MC Liu CM Chiu YN Hsieh MH Hwang TJ. Follow-up of subjects with suspected pre-psychotic state in Taiwan. Schizophr Res. (2011) 126:65–70. 10.1016/j.schres.2010.10.028 21112187

[49] Leucht S Samara M Heres S Davis JM. Dose equivalents for antipsychotic drugs: the DDD method. Schizophr Bull. (2016) 42(Suppl. 1):S90–4. 10.1093/schbul/sbv167 27460622 PMC4960429

[50] Liu CC Hwu HG Chiu YN Lai MC Tseng HH. Creating a platform to bridge service and research for early psychosis. J Formos Med Assoc. (2010) 109:543–9. 10.1016/S0929-6646(10)60089-7 20654794

[51] Liu CC Takeuchi H. Achieving the lowest effective antipsychotic dose for patients with remitted psychosis: a proposed guided dose-reduction algorithm. CNS Drugs. (2020) 34:117–26. 10.1007/s40263-019-00682-8 31741178

[52] Tani H Takasu S Uchida H Suzuki T Mimura M Takeuchi H. Factors associated with successful antipsychotic dose reduction in schizophrenia: a systematic review of prospective clinical trials and meta-analysis of randomized controlled trials. Neuropsychopharmacology. (2020) 45:887–901. 10.1038/s41386-019-0573-7 31770770 PMC7075912

[53] Uchida H Suzuki T Takeuchi H Arenovich T Mamo DC. Low dose vs standard dose of antipsychotics for relapse prevention in schizophrenia: meta-analysis. Schizophr Bull. (2011) 37:788–99. 10.1093/schbul/sbp149 19946012 PMC3122280

[54] Mayoral-van Son J de la Foz VO Martinez-Garcia O Moreno T Parrilla-Escobar M Valdizan EM. Clinical outcome after antipsychotic treatment discontinuation in functionally recovered first-episode non-affective psychosis individuals: a 3-year naturalistic follow-up study. J Clin Psychiatry. (2016) 77:492–500. 10.4088/JCP.14m09540 26759992

[55] Horowitz MA Murray RM Taylor D. Tapering antipsychotic treatment. JAMA Psychiatry. (2020) 78:125–6. 10.1001/jamapsychiatry.2020.2166 32777027

[56] Chouinard G Samaha AN Chouinard VA Peretti CS Kanahara N Takase M. antipsychotic-induced dopamine super sensitivity psychosis: pharmacology, criteria, and therapy. Psychother Psychosom. (2017) 86:189–219. 10.1159/000477313 28647739

[57] Tsuboi T Suzuki T Bies RR Remington G Pollock BG Mimura M. Challenging the need for sustained blockade of dopamine D(2) receptor estimated from antipsychotic plasma levels in the maintenance treatment of schizophrenia: a single-blind, randomized, controlled study. Schizophr Res. (2015) 164:149–54. 10.1016/j.schres.2015.03.025 25864950

[58] Mizuno Y Bies RR Remington G Mamo DC Suzuki T Pollock BG. Dopamine D2 receptor occupancy with risperidone or olanzapine during maintenance treatment of schizophrenia: a cross-sectional study. Prog Neuropsychopharmacol Biol Psychiatry. (2012) 37:182–7. 10.1016/j.pnpbp.2011.12.013 22230651

[59] Moriguchi S Bies RR Remington G Suzuki T Mamo DC Watanabe K. Estimated dopamine D(2) receptor occupancy and remission in schizophrenia: analysis of the CATIE data. J Clin Psychopharmacol. (2013) 33:682–5. 10.1097/JCP.0b013e3182979a0a 23899638

[60] Takeuchi H Suzuki T Bies RR Remington G Watanabe K Mimura M. Dose reduction of risperidone and olanzapine and estimated dopamine D(2) receptor occupancy in stable patients with schizophrenia: findings from an open-label, randomized, controlled study. J Clin Psychiatry. (2014) 75:1209–14. 10.4088/JCP.13m08841 25099201

[61] Uchida H Suzuki T Graff-Guerrero A Mulsant BH Pollock BG Arenovich T. Therapeutic window for striatal dopamine D2/3 receptor occupancy in older patients with schizophrenia: a pilot PET study. Am J Geriatr Psychiatry. (2014) 22:1007–16. 10.1016/j.jagp.2013.01.045 25217025

[62] Takeuchi H Suzuki T Remington G Bies RR Abe T Graff-Guerrero A. Effects of risperidone and olanzapine dose reduction on cognitive function in stable patients with schizophrenia: an open-label, randomized, controlled, pilot study. Schizophr Bull. (2013) 39:993–8. 10.1093/schbul/sbt090 23821768 PMC3756793

[63] Zhou Y Li G Li D Cui H Ning Y. Dose reduction of risperidone and olanzapine can improve cognitive function and negative symptoms in stable schizophrenic patients: a single-blinded, 52-week, randomized controlled study. J Psychopharmacol. (2018) 32:524–32. 10.1177/0269881118756062 29493377

[64] Kim JS Baek JH Choi JS Lee D Kwon JS Hong KS. Diagnostic stability of first-episode psychosis and predictors of diagnostic shift from non-affective psychosis to bipolar disorder: a retrospective evaluation after recurrence. Psychiatry Res. (2011) 188:29–33. 10.1016/j.psychres.2010.09.017 21056477

[65] Fusar-Poli P Cappucciati M Rutigliano G Heslin M Stahl D Brittenden Z. Diagnostic stability of ICD/DSM first episode psychosis diagnoses: meta-analysis. Schizophr Bull. (2016) 42:1395–406. 10.1093/schbul/sbw020 26980142 PMC5049518

[66] Zavos HM Eley TC McGuire P Plomin R Cardno AG Freeman D. Shared etiology of psychotic experiences and depressive symptoms in adolescence: a longitudinal twin study. Schizophr Bull. (2016) 42:1197–206. 10.1093/schbul/sbw021 26994398 PMC4988737

[67] McGorry P Alvarez-Jimenez M Killackey E. Antipsychotic medication during the critical period following remission from first-episode psychosis: less is more. JAMA Psychiatry. (2013) 70:898–900. 10.1001/jamapsychiatry.2013.264 23824206

[68] Liu CC Hsieh MH Chien YL Liu CM Lin YT Hwang TJ. Protocol of guided antipsychotic reduction to reach minimum effective dose (GARMED) in patients with remitted psychosis based on pragmatic design. Early Interv Psychiatry. (2021) 10.1111/eip.13144. [Epub ahead of print]. 33751764

[69] Murray RM Di Forti M. Increasing expectations and knowledge require a more subtle use of prophylactic antipsychotics. World Psychiatry. (2018) 17:161–2. 10.1002/wps.20517 29856571 PMC5980442

[70] Leucht S. Is there compelling evidence that schizophrenia long-term treatment guidelines should be changed? World Psychiatry. (2018) 17:166–7. 10.1002/wps.20520 29856564 PMC5980526

